# Evaluation of End-of-Life Reverse Osmotic Membrane for High-Retention Anaerobic Membrane Bioreactor

**DOI:** 10.3390/membranes15110323

**Published:** 2025-10-22

**Authors:** Oriol Morató Torras, Hiren D. Raval, Bianca Zappulla-Sabio, Ignasi Rodriguez-Roda, Hèctor Monclús, Gaetan Blandin

**Affiliations:** 1 LEQUIA, Institute of the Environment, University of Girona, 17003 Girona, Spain; orimoto01@gmail.com (O.M.T.); bianca.zappulla@udg.edu (B.Z.-S.); ignasi.rodriguezroda@udg.edu (I.R.-R.); hector.monclus@udg.edu (H.M.); 2 Membrane Science and Separation Technology Division, CSIR-Central Salt and Marine Chemicals Research Institute, Gijubhai Badheka Marg, Bhavnagar 364002, Gujarat, India; hirenraval@csmcri.res.in

**Keywords:** anaerobic membrane bioreactor, granular biomass, end-of-life reverse osmosis membrane, ultrafiltration, nanofiltration

## Abstract

Following on from a circular economy in water, membrane technologies can play a role in resource recovery and high-quality water production but should also consider membrane industry circularity. Anaerobic membrane bioreactors (AnMBRs) are being used for advanced wastewater treatment, and their applications are growing due to advantages like lower sludge volume, better permeate quality, and the generation of biogas. High-Rejection (HR) AnMBRs retain a higher fraction of dissolved and particulate components to further promote resource recovery and obtain improved effluent quality. With the development of membrane technologies, end-of-life (EOL) membrane recycling is emerging for various applications. The feasibility of transforming EOL Reverse Osmosis (RO) membranes into ultrafiltration (UF)- and nanofiltration (NF)-like membranes and applying these membranes to submerged HR-AnMBR applications was evaluated. A small pilot AnMBR with granular biomass was operated with EOL RO membranes converted to submerged UF- and NF-like membranes and compared to commercial microfiltration (MF) membranes. UF- and NF-like plates were constructed, characterized, and introduced step-by-step into the AnMBR by the substitution of MF plates. A chemical oxygen demand (COD) removal study showed that while 77% removal of COD was possible with MF membranes, improved COD removal (i.e., 81.40% and 88.39%) was achieved using UF-like and NF-like membranes, respectively. Because of the higher retention of salts of the NF-like membrane, the salinity in the membrane bioreactor increased from 1300 to 1680 µS·cm^−1^ but stabilized quickly and without a negative impact on system performance. Even without cleaning, minimal fouling and flux decline were observed for all tested configurations thanks to the use of granular biomass and low permeation flux. Permeate flux in the case of the NF-like membrane was slightly lower due to the required higher pressure. The present study demonstrated that the EOL-RO membranes may find applications in HR-AnMBRs to achieve superior permeate quality and move toward circular membrane processes.

## 1. Introduction

With growing population and industrial production, there is an equivalent increase in wastewater (WW) treatment and reuse plants, as well as desalination plants, where membrane technologies play a major role. Within the context of a circular economy, membrane technologies are crucial (1) to enable water reuse and recycling, (2) to recover resources from wastewater, (3) to minimize energy, and also (4) to integrate the sustainable management of membrane materials, especially by extending their life span and moving toward membrane recycling.

A typical integration of membrane technologies within WW treatment processes is the development of a membrane bioreactor, which integrates selective membranes within biological reactors developed since the 1960s and nowadays state-of-the-art technology [1]. Then, in the 1980s, anaerobic membrane bioreactors (AnMBRs) were developed, allowing the transformation of WW organic matter into biogas [2]. AnMBRs present several advantages over MBRs, such as lower sludge volume, better quality of effluent treated, biogas production, and the potential to produce energy [3]. However, their development remained mostly limited to high chemical oxygen demand (COD) streams such as industrial WW, since with urban WW, the relatively low COD content limits the methane production, which does not offset the energy demand from membrane operation (biogas sparging, heating, and permeate pump) [4]. Recently, granular biomass-based AnMBRs (G-AnMBRs) have gained interest since granules boost biomass activity, increase microbial diversity, improve resistance to shocks, and reduce fouling [5]. Recent works have demonstrated the possibility of operating G-AnMBRs for domestic WW at psychrophilic (ambient) temperatures and to achieve high COD removal, increase effluent quality, and lead to a net energy balance thanks to biogas production [6]. Thus, G-AnMBRs present key advantages for broader implementation, especially in domestic WW treatment for water reuse.

Anaerobic digestion involves four different steps: hydrolysis, acidogenesis, acetogenesis, and methanogenesis [7]. Hydrolysis involves the transformation of insoluble and complex organic materials mediated by enzymes. This process converts higher-molecular-weight compounds such as polysaccharides, lipids, proteins, fats, etc., into soluble organic materials. Then, the soluble compounds are degraded further into short-chain organic acids such as propanoic acids, butyric acids, acetic acids, alcohols, hydrogen, carbon dioxide, etc. (acidogenesis). During acetogenesis, the products are converted to methanogenic substrates like acetate, hydrogen, and carbon dioxide. In the last step (methanogenesis), the substrates are converted to methane and carbon dioxide [8,9]. Anaerobic bacteria grow more slowly than aerobic bacteria, and thus, the retention of biomass in the reactor is critical for the operation. AnMBRs can partially address this problem by retaining micro-organisms and biomass within the tank. Thus, the retention of biomass is very important in the anaerobic process; membrane type and design play a key role. Two configurations of AnMBR are the most popular and widely practiced: external (or side-stream) AnMBRs and submerged AnMBRs. While external AnMBRs offer the advantages of better fouling control and ease of operation and replacement, the energy consumption is higher in the order of 10 KWH/m^3^ [10]. Moreover, the higher the cross-flow velocity, the poorer the response of the biomass [11,12]. Thus, submerged AnMBRs are relevant and of interest for lower energy consumption and better control of operations with the reactor.

Membranes used in MBRs are porous membranes (i.e., microfiltration (MF) or ultrafiltration (UF)), which allow the rejection of suspended solids, macromolecules such as proteins, and some pathogens but are not efficient for removing smaller molecules such as salts, pesticides, or pharmaceuticals, which are of high concern in the context of water reuse [2]. As a step forward toward AnMBR systems, high-retention membrane bioreactor (HR-MBR) systems were developed using dense membranes such as nanofiltration (NF) membranes, osmotic MBRs, or membrane distillation MBRs [13,14]. Organic contaminants can be effectively retained because of the higher degree of rejection by dense membranes, thereby elongating their retention time in the bioreactor and enhancing their degradation [15,16]. Therefore, HR-MBRs can offer an option to produce an improved quality of treated effluent.

There are several challenges associated with the application of HR-MBRs. One main concern is the salinity build-up because of the high-rejection membranes used in the system; the different salts are also being rejected, and thus, they stay in the membrane bioreactor. The physical and biochemical properties of activated sludge change, e.g., probable effects are an increased concentration of extracellular polymeric substances and soluble microbial products in the bioreactor [17,18]. Higher concentrations of extracellular polymeric substances and soluble microbial products could yield unfavorable physicochemical conditions for oxygen transfer and increased membrane fouling [19]. The use of membranes that are semi-selective to salts, like NF membranes, may limit salinity build-up and allow for more sustainable operation. The use of NF membranes, with lower water permeability, typically requires higher operating pressure and/or leads to lower permeation flux. Thus, operating an HR-MBR and defining the optimum permeability assures high selectivity, and sustainable permeation flux is a trade-off. Membrane degradation and the compatibility of an HR MBR into a biological reactor is also a key challenge. The biological degradation and hydrolysis of a membrane, i.e., cellulose acetate, in an NF-MBR system was observed after long-term operation [20]. Such behavior has not been observed with polyamide (PA)-based membranes, but it is still to be further studied. Fouling propensity and mechanisms are also still to be further evaluated since PA membranes were found to be more prone to fouling [1]. A study comparing UF and NF MBRs demonstrated that different fouling mechanisms were observed, i.e., pore-blocking fouling being the main reason for UF membrane fouling, while NF-MBR fouling was dominated by cake-layer fouling, leading to transmembrane pressure increase [21].

In parallel, desalination plants and reverse osmosis (RO) membranes have gained significant attention due to their high efficiency in removing salts and other impurities from seawater. However, the rapid increase in desalination capacity to ensure a reliable and safe water supply has led to a drastic rise in the use of RO membranes. A major challenge of this technology is the relatively short lifespan of RO membranes, typically around five years, after which they are discarded. In 2022, approximately 35,000 tons of end-of-life (EOL) RO membranes were used, and by 2025, more than 2 million modules are expected to be disposed of [22]. Membranes are generally replaced once their performance (permeability and rejection coefficients) declines significantly and beyond recovery, or simply because they reach the useful lifespan advised by the manufacturers, or because financing for membrane replacement is approved. The pressing need for more sustainable practices has driven research into the feasibility of recycling EOL RO membranes. EOL RO membrane recycling options include upcycling to prepare the membrane with improved sieving efficiency, downcycling to prepare the membrane with lower sieving accuracy, membrane refabrication with recovered material from the EOL RO membrane, and utilizing parts of the membrane elements for different applications [23]. The recycling and rejuvenation of thin-film composite (TFC) RO membranes offers the possibility to extend the lifespan of the membrane, at the same time addressing the problem of its disposal. Most studies are focused on the downcycling approach and the transformation of RO membranes into UF- or NF-like membranes. Studies reported (Table 1) that sodium hypochlorite allowed for the downcycling of the membranes by controlling the partial or total degradation of the polyamide layer to convert them into NF-/UF-like membranes by playing on the contact time and dose [24].

Transformed membranes were tested for several applications, especially in WW treatment [32,33,34]. It has been reported that RO membranes at the end of their life can be converted to UF-like membranes with 99.9997% removal of *E. coli* bacteria [35]. This opens new pathways for the application of EOL RO membranes. Gravity-driven systems using RO modules converted to NF-/UF-like ones were operated at low pressure to avoid the grid-based power, and Log 4 and Log 5 removal of bacteria for UF-like and NF-like membranes, respectively, were achieved [36,37].

While several applications of recycled membranes have been attempted, the application of recycled/transformed membranes in a submerged anaerobic membrane bioreactor is a research gap. This study proposes to evaluate the potential to use EOL RO membranes transformed into UF- and NF-like membranes for potential application in HR Granular AnMBRs as a potential concept to tackle sustainability in water and membrane processes, moving toward circularity. First, submerged modules based on EOL membranes were constructed and characterized. Then, UF and NF module performance in terms of COD removal and sustainable operation were assessed and compared to a commercial MF module, considering COD removal, operating pressure, salinity build-up, fouling, and cleaning.

## 2. Materials and Methods

### 2.1. Membranes, Modules, and Characterization

**Figure 1:**
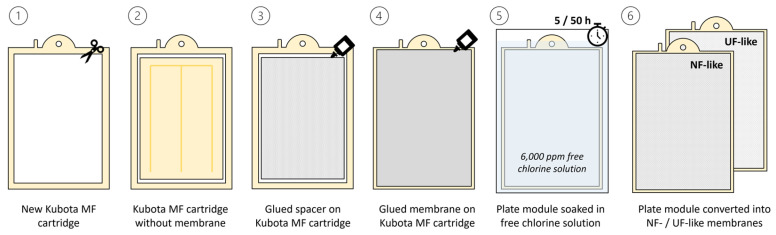
Step-by-step protocol for submerged membrane modules fabrication and transformation from existing commercial MF plates to NF-/UF-like membrane plates.

Kubota MF cartridge 203 flat sheet modules in Polyethylene Terephthalate (PET) and Chlorinated Polyethylene (PE-C) with a nominal pore size of 0.4 μm, 0.1 m^2^ surface area, and 6 mm cartridge width were acquired from KUBOTA Membrane Europe (UK). A new commercial RO membrane module, SW30HRLE, was purchased from DWS Octochem, DuPont (Vandalia, IL, USA). The module was opened, and the membranes were cut into coupons using a standardized cutting pattern. Similarly, a SW30HRLE-400 module, sourced from an industrial application and referred to here as SW30-used, was opened in the laboratory, and membrane sheets were cut following the same pattern. SW30 and SW30-used membranes were glued onto Kubota MF cartridge 203 frames after removing the MF membrane to build plate and frame modules (Figure 1). A 6000 ppm free chlorine solution was prepared from sodium hypochlorite (NaClO) purchased from Sigma Aldrich (Barcelona, Spain). Plates were immersed in a 6000 ppm free chlorine solution at ambient temperature (20 °C) and pH 7 for 5 and 50 h to achieve the total dose of 30,000 and 300,000 ppm·h, respectively, to convert the SWRO membranes into NF-like (R-NF) and UF-like membranes (R-UF), respectively [24,38]. A schematic protocol of the module production is presented in Figure 1. The impact of free chlorine on membrane properties was assessed through permeability and rejection tests as described hereafter.

Membrane properties were assessed using the same reactor as used by Sanchez et al. [39]. The reactor was filled with 4 L of water. Permeate extraction was conducted using a peristaltic pump (Watson Marlow, Falmouth 323S, UK) through negative pressure (suction) operated successively at 33, 66, and 99 rpm to allow for a permeation flux of 5, 10, and 15 L/m^2^/h, respectively. Air sparging was provided through three drilled holes (1 mm diameter) below the membrane module, distributed along the length, and controlled by a gas flowmeter at flow rates of 25 L/h. A pressure sensor was connected to the permeate line to measure transmembrane pressure (TMP), and permeate was collected in a tank placed on a Kern EWJ balance to measure flux and permeability. Membrane plate permeabilities were tested first using DI water and then using the wastewater (WW) of the AnMBR. SW30 and SW30-used membrane modules were compared. Similar behavior was observed for used and new membranes; thus, only plates made from SW30 new modules were later used for AnMBR operation.

SW30 and SW30-used membrane surfaces before and after conversion were characterized through a Field-Emission Scanning Electron Microscope (FE-SEM) and Fourier Transform Infrared (FTIR) characterizations following similar procedures as described in our former study [40]. FTIR spectroscopy analysis was conducted using a PerkinElmer Spectrum 100 FT-IR instrument (PerkinElmer, Shelton, CT, USA) equipped with a 45° multi-reflection ZnSe flat plate crystal as the Attenuated Total Reflectance (ATR) element. FE-SEM membrane surface analysis was conducted using a NanoSEM 230 instrument, Thermo Fisher Scientific, Bothell, WA, USA.

### 2.2. Pilot-Scale G-AnMBR Setup and Operating Conditions

**Figure 2:**
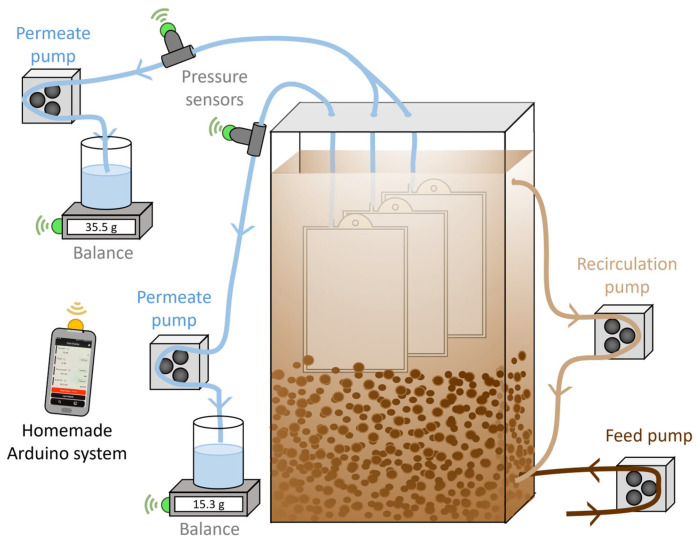
Pilot-scale G-AnMBR setup scheme composed of a 17 L tank, recirculation pump, permeate pump, and feed pump. TMP and permeate flow rates were recorded by pressure sensors and a balance on permeate lines connected by Bluetooth to an Arduino-based app.

The pilot-scale (Figure 2) and operating conditions were similar to those used in our former study [18] and featured a rectangular reactor (282 × 100 × 900 mm) with a working volume of 17 L. Up to 3 flat sheet membrane modules with a filtration surface area of 0.1 m^2^ each were placed in the reactor. Modules were operated under negative pumping pressure using 323S peristaltic pumps (Watson-Marlow, Falmouth, UK) without relaxation or gas sparging. Permeate fluxes were controlled by the pump velocity and were monitored by the increase in mass in permeate tanks using a Kern EWJ balance. Except for specific conditions, the average targeted permeation flux was 3.3 L/m^2^/h.

The reactor was seeded with 87 g total suspended solids (TSS)/L of already formed anaerobic granular sludge obtained from a G-AnMBR pilot operated at Institut Européen des Membranes (University of Montpellier, France) [6] with a volatile fraction of 57% and a particle size distribution of mostly above 1 mm (>85%) [39]. The reactor was fed with synthetic WW that was prepared and stored in a 150 L stirred metallic tank cooled at 6 °C with a similar composition as used in our former study [18]. The feed COD concentration was 500 mg/L. The hydraulic retention time (HRT) was set at 17 h with the aim of achieving an optimal organic matter removal of 90% [6]. All experiments were conducted at room temperature, 20 ± 2 °C. The biomass level filled the bottom part of the reactor up to the bottom part of the membrane modules. A recirculation pump set at 40 L/h was used to assure good contact between the WW and the biomass and a slight microbial granule fluidization.

The pilot was fully monitored by a homemade Arduino system [41]. Conductivity, temperature, and pH sensors were placed in the G-AnMBR supernatant. Transmembrane pressure was measured using a pressure sensor in the permeate line. All sensors were Arduino-compatible and purchased from DF Robot (Shanghai, China). WW was fed constantly to the reactor to maintain the level of WW in the reactor above the membranes.

### 2.3. Operation of the G-AnMBR

The G-AnMBR was operated first for one week in a configuration with 100% MF (using 3 MF membrane modules). Then, one MF module was substituted by an EOL membrane transformed into a UF module and maintained for at least one week in this new operating mode. Step by step, MF modules were substituted first by UF modules. Thus, the reactor was successively operated with various MF/UF extraction ratios, i.e., 100% MF, 2/3 MF-1/3 UF, 1/3 MF-2/3 UF, and 100% UF. A similar procedure was used with EOL membranes converted to NF membranes substituting MF membranes. In total, the reactor was operated for 7 weeks, each corresponding to a specific configuration (Table 2). The step-by-step substitution of membranes allowed for the progressive adaptation of biomass and G-AnMBR operation to changing conditions and for the comparison of 2 types of membrane in strictly similar biomass conditions.

Membrane modules were operated at constant flux, and therefore, fouling occurrence was assessed through TMP increase. Membranes were typically cleaned when a severe increase in TMP was observed (increase in TMP by 0.3 bar and/or decrease in permeation flux by 25%) and before changing the configuration. Cleaning consisted (1) flushing the fouling layer using 1 L of DI water. During each step, chemical oxygen demand (COD) analyses were realized every 2 days to check the organic matter removal, taking samples from the feed, reactor, and permeate and using Lovibond kits (COD Vario Tube Test 0–1500 mg/L and 0–150 mg/L) and a spectrophotometer (Photometer-System MD100, Tintometer GmbH, Lovibond Water Testing, Dortmund, Germany).

## 3. Results and Discussion

### 3.1. EoL Membrane Conversion-Membrane Properties

**Figure 3:**
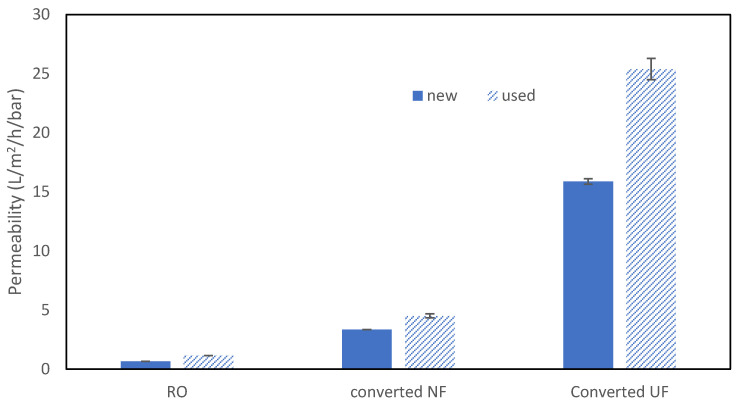
Comparison of the permeability of new and used RO membranes converted to NF and UF membranes.

As can be seen in Figure 3, the permeabilities of original RO membranes were very low, with slightly higher permeability for used RO membranes, probably resulting from degradation from their previous industrial usage. After conversion by soaking in 30,000 ppm·h of sodium hypochlorite, membranes were transformed to NF-like membranes with a significant increase in permeability, i.e., 3.4 and 4.5 L/m^2^/h/bar for new and used RO membranes, respectively. Longer soaking with doses of 300,000 ppm·h in sodium hypochlorite further increased permeability up to 16 and 25 L/m^2^/h/bar for new and used RO membranes, respectively. Those numbers are in the range (lower range) of usually observed permeabilities for membranes transformed into NF and UF membranes using similar doses of NaOCl [24,28]. The slightly lower permeability observed could be attributed to (1) higher tolerance of SW30 membranes to NaOCl [24,28,30] and the use of a submerged setup and therefore very low operating pressure used in our evaluation setup for submerged plates (below 1 bar, suction mode).

**Figure 4:**
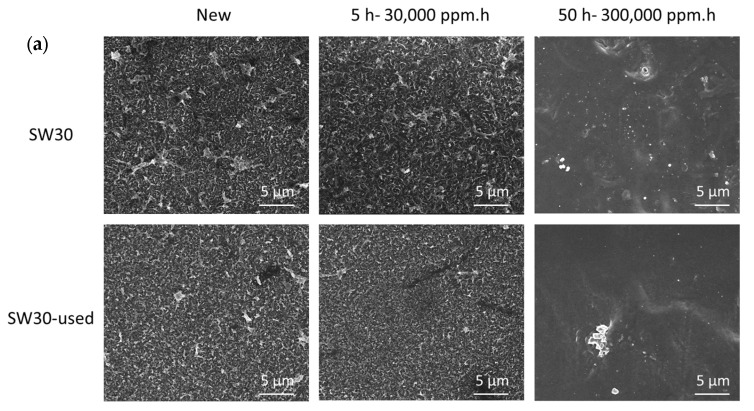

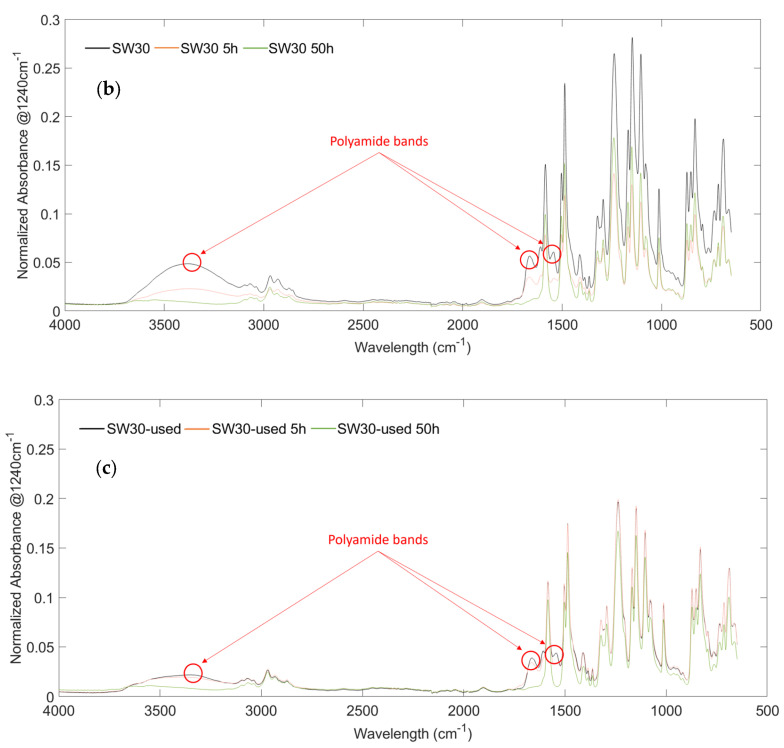
(**a**) SEM characterization of new and used SW30 membranes before and after exposure to 30,000 (5 h) and 300,000 ppm·h (50 h) free chlorine for conversion to NF and UF membranes, respectively. FTIR spectra for the studied membranes of (**b**) SW30 and (**c**) SW30-used before and after conversion.

As observed through FE-SEM and FTIR characterization (Figure 4), exposure to free chlorine led to a progressive degradation and removal of the polyamide layer, especially visible after 300,000 ppm·h (absence of ridge and valley structure typical of polyamide layer in SEM and a reduction in typical polyamide bands (3300, 1650, and 1550 cm^−1^)). As such, partial and total degradation of the PA layer after 30,000 and 300,000 ppm·h doses, respectively, was confirmed as described in our previous study using similar membranes [40].

**Figure 5:**
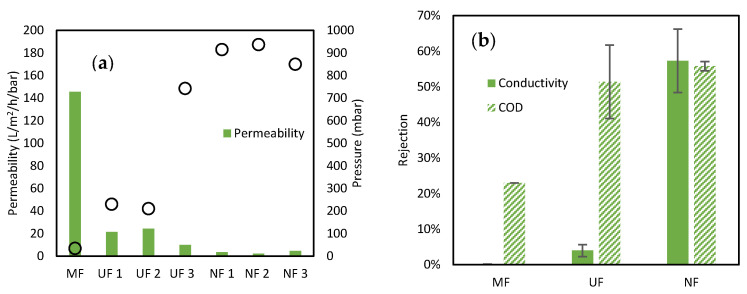
(**a**) Permeability and operating pressure and (**b**) rejection of conductivity and COD of MF, UF, and NF membranes.

Further characterization tests were then conducted using WW to evaluate the initial properties of the MF and three R-UF and R-NF to be used in the G-AnMBR. Operating pressure, permeability, and rejection in terms of conductivity and COD are presented in Figure 5a,b.

MF membranes present a permeability of 145.6 L/m^2^/h/bar, well below the typical permeability usually observed for this membrane [39]. Such behavior can be attributed to the operating conditions used in our setup, i.e., very low flux (5 L/m^2^/h) in comparison with typical fluxes applied for the evaluation of MF membranes and the very low observed resulting suction pressure. However, those conditions were chosen to be close to the operating pressure that will be used in AnMBRs and therefore are representative of membrane permeability in this application. As expected, and observed previously, NF and UF featured much lower permeabilities than MF membranes. Permeabilities of the three UF membranes varied from 9.8 to 24.3 L/m^2^/h/bar, attesting that even using similar membranes and similar conversion conditions, final variations can occur, probably resulting from the initial coupon variability or potential partial drying within the process [42]. Similar performance was observed for all three UF membranes, with very low conductivity (salts) rejection, confirming that the PA layer of the RO membranes (transformed to UF) was fully removed. The main difference is the resulting operating pressure required to reach the expected flux of 5 L/m^2^/h, significantly higher for UF3 than for the two other UF membranes due to its lower permeability (Figure 5a).

Regarding NF membranes, permeability variation in the range of 2.1 to 4.5 L/m^2^/h/bar was also observed. However, in all cases, even with such low permeability, it was possible to obtain 5 L/m^2^/h while operating below 1000 mbar, confirming the potential to use NF membranes in submerged mode operated with limited transmembrane pressure [20,43]. An important rejection capability of NF membranes was observed both regarding salts and COD (Figure 5b). Very different characteristics regarding rejection were confirmed when comparing MF, UF, and NF membranes with (1) MF featuring no rejection of salts and partial–low rejection of COD, (2) UF showing low/no rejection of salts but significant rejection of CDO, and (3) NF confirming superior rejection of both conductivity and COD. Those numbers confirm the expected properties of the transformed membranes and their potential to be used for operation in HR AnMBRs.

### 3.2. G-AnMBR Operation

#### 3.2.1. Flux and Operating Pressure

**Figure 6:**
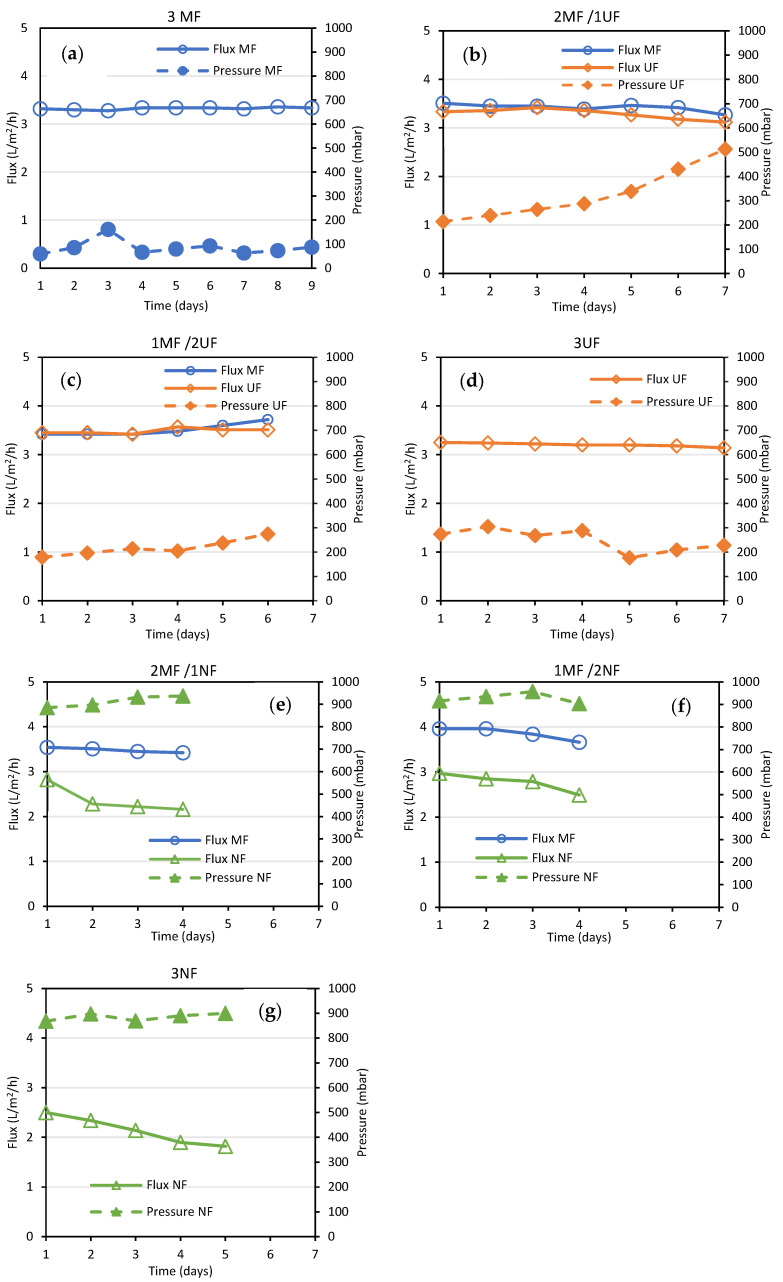
Flux and pressure observed during filtration by MF, UF, and NF for each sequence, i.e., (**a**) 3 MF membranes, (**b**) 2 MF and 1 UF, (**c**) 1 MF and 2 UF, (**d**) 3 MF, (**e**) 2 MF and 1 NF, (**f**) 1 MF and 2 NF, (**g**) 3 NF.

The G-AnMBR was initially seeded with granular biomass and operated for 3 weeks until reaching stable operation (stable COD rejection and clean supernatant). Then, the system was monitored for operation, with three MF plates cleaned before usage. As observed in Figure 6a, stable operation was observed with stable flux and operating pressure below 100 mbar. Only after day 3, cleaning was performed (flushing with water) due to a slight increase in the transmembrane pressure resulting from the unwanted lifting of some biomass and the release of extrapolymeric substance on the membrane surface following an issue with the recirculation loop. Starting from day 4, the system was operated for 6 days without the need for additional cleaning. Then, MF membranes were replaced one by one by UF membranes; the results of each sequence are presented in Figure 6b–d. Similar fluxes were observed with UF membranes without significant changes during the weeks of operation. The operating pressure of UF was systematically higher (200–300 mbar) than that observed for MF, resulting from the lower permeability of UF membranes as described earlier. Overall, no major increase in UF operation pressure was observed even when operating with solely UF membranes, except for the configuration of 2 MF/1 UF, where the pressure increased up to 500 mbar after 7 days. This behavior was attributed to a mixing of the biomass when opening the reactor and substituting the membranes, since the supernatant appeared more charged after this operation. Later, stronger attention was paid to limiting unwanted mixing during the changing or cleaning of the membranes. Overall, stable operation was observed in a system operated with neither gas sparging nor relaxation during one week of operation, confirming very low fouling propensity when working with G-AnMBRs. No stronger fouling behavior was observed regarding the recycled UF membrane in comparison with the NF one within the limited time of the study.

Operation with NF membranes resulted in distinct behavior (Figure 6e–g) with regard to fluxes and pressure. For each sequence, the system could not be operated for more than 4 to 5 days due to flux decrease in the reactor, which may impact the overall HRT and the repartition of permeates extracted through MF and NF membranes, respectively. On the one hand, MF membranes did not show any modification with constant operation at 3.3 L/m^2^/h, but on the other hand, fluxes in NF were lower than expected at the beginning of each step (limited to 3 L/m^2^/h and furthermore decreasing with time). This is a result of the observed pressure in the NF permeate line, close to 1000 mbar, which is a limiting parameter for the proper operation of the peristaltic pump. On the one hand, this highlights the potential to operate HR-AnMBRs with recycled NF membranes, but on the other hand, like for any other HR-MBR system, pressure might be a limitation, and a system operating at very low fluxes or under higher transmembrane pressure should be designed [13,43]. Even if a noticeable decrease in filtration performance was observed, it remains minimal in comparison with our previous study, where the integration of FO membranes was assessed and therefore confirms the potential to integrate NF membranes in G-AnMBR systems [18]. The higher fouling propensity of NF membranes was already observed in other HR-MBR studies, most likely because of the high rejection of extrapolymeric substances (EPSs) and soluble microbial products (SMPs).

#### 3.2.2. COD Removal and Conductivity

**Figure 7:**
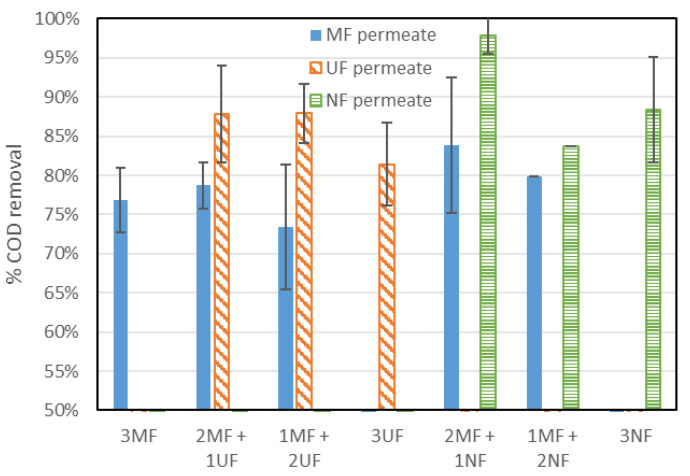
COD removal for each membrane configuration.

Several samples were taken to calculate COD removal for each configuration and better assess the impact of each membrane on the overall performance of the system (Figure 7). With MF membranes, COD removal was limited to 80%, which is in line with our former study using similar conditions [18]. With UF and NF membranes, higher rejections were observed, i.e., in the ranges of 84.4–89% and 83.8–97.9% for UF and NF membranes, respectively. This was expected since higher rejection of COD was observed for those membranes (Figure 5b). Moreover, the use of higher rejection membranes is also interesting for increasing the residence time of COD in the G-AnMBR and therefore further improving its degradation, as observed for other HR-MBRs [13]. The slight increase in COD removal observed in the MF permeate line when the G-AnMBR was operated in mixed operation (MF-UF or MF-NF) reinforces this statement in line with results from former studies showing that high-retention MBRs can allow for advanced degradation of organic contaminants [13,18,43,44]. This effect is somehow limited with the UF and NF membranes tested in this study, since they offer only partial rejection to the colloidal or dissolved COD used in the synthetic WW.

**Figure 8:**
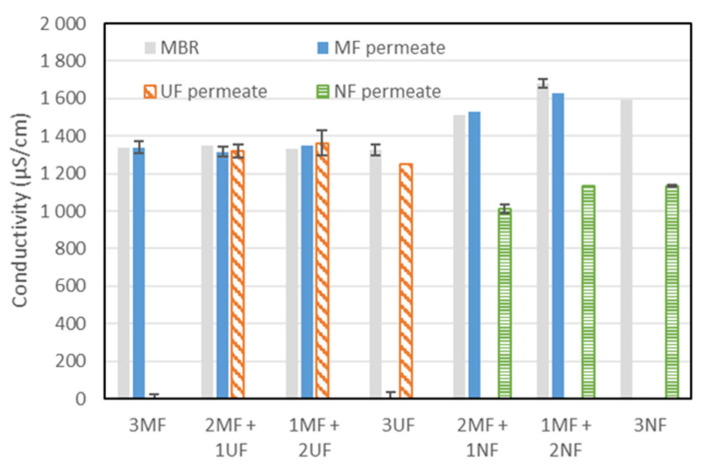
Conductivity in the AnMBR and permeates for each tested membrane configuration.

Conductivity was also assessed during each set of experiments. Conductivities observed in MF and UF permeates (1300 µS/cm) were similar to those observed in MBR mixed liquor (Figure 8). Operation under 100% MF, 100% UF, or mixed UF/MF operation did not lead to any change with regard to conductivity in mixed liquors or permeates. Such behavior was expected since both MF and UF membranes are not selective to salts. Conversely, following the integration of NF membranes, changes were observed in terms of conductivity in the MBR and in the produced permeates. Due to the partial rejection of salts by NF membranes, the lowest conductivity was measured in NF permeate (down to 1000 µS/cm). Consequently, salinity accumulated in the MBR mixed liquor with an increase from 1300 (in the previous sequence with three UF) up to 1680 µS/cm. The accumulation of salt was partly compensated by their leaching via MF membranes, which are not selective to salts, as already reported for MBRs operated simultaneously by salt-selective (forward osmosis) membranes and MF/UF membranes [45,46]. However, when operating only with NF membranes, unlike that observed in other studies with salt-selective membranes, no additional increase in conductivity was observed. This results from the partial rejection of NF membranes, leading to an equilibrium. Basically, partial rejection of NF membranes leads to an increase in salinity within the AnMBR (around 1600 µS/cm), well above the feed salinity of 1250 µS/cm, but due to partial rejection, conductivity in the NF permeates reaches 1200 µS/cm, like feed WW conductivity, and therefore reaches equilibrium.

## 4. Conclusions

This work aimed at exploring applications of recycled membranes for membrane bioreactor applications. The transformed membranes were successfully deployed for G-AnMBR applications. RO membranes were transformed and operated both as NF (nanofiltration) and UF (ultrafiltration) modules for AnMBR applications. Stable operation was achieved, although pressure management remained a key operational factor, especially when considering NF membranes. Fouling did not present a significant challenge, thanks to the use of granular biomass. UF membranes allowed for increased COD rejection, while NF membranes achieved even higher COD rejection and enhanced salinity retention. With NF membranes, an increase in salinity was observed in the bioreactor because of partial salinity rejection by this membrane, but it stabilized quickly and did not have a significant impact on overall operation. Thus, the present study shows that the EOL RO membranes may be used effectively for HR-AnMBR applications. The future scope of work may include studies on biogas production, the characterization of biomass with increased salinity, the integration of biogas scouring, and the fate of emerging contaminants.

## Figures and Tables

**Table 1:** Studies using sodium hypochlorite to convert end-of-life reverse osmosis membranes into nanofiltration-/ultrafiltration-like membranes.

Initial Membrane	Concentration (ppm)	Time (h)	Dose (ppm·h)	Final Membrane	Permeability (LMH/bar)	Salt Rejection (%)	Ref.
RO	-	2–5	220,000–550,000	UF	50.4	11.6	[25]
RO	75,000	4	300,000	UF	79.1	16.7	[26]
RO	13,000	18.5	240,500	UF	51	0	[27]
New BW30	125,000	2.4	300,000	UF	59	<1	[28]
EoL BW	125,000	2.4	300,000	UF	115	<1	
EoL BW	125,000	2.4	300,000	NF	9	<1	
EoL BW30	124	242	30,008	NF	40.6	1.7	[24]
EoL BW	124	242	30,008	NF	37.4	1.5	
EoL SW30	124	242	30,008	NF	33.8	4.6	
EoL SW	124	242	30,008	NF	11	3.6	
EoL BW30	55,000	5.4	297,000	UF	116.7	12.6	[29]
EoL BW	6000	50	300,000	UF	86.1	-	[30]
EoL BW	6000	5	30,000	NF	14	-	
EoL BW30	6000	50	300,000	UF	59.6	-	
EoL BW30	6000	5	30,000	NF	5.8	-	
RO	4000	13	52,000	NF	80	25.5	[31]

**Table 2:** Sequences of operation of a G-AnMBR with different membrane systems.

Week						
1	2	3	4	5	6	7
3 MF						
	2 MF + 1 UF					
		1 MF + 2 UF				
			3 UF			
				2 MF + 1 NF		
					1 MF + 1 NF	
						3 NF

## Data Availability

The raw data supporting the conclusions of this article will be made available by the authors on request.
